# The protein tyrosine phosphatase Lyp/PTPN22 drives TNFα-induced priming of superoxide anions production by neutrophils and arthritis

**DOI:** 10.1016/j.freeradbiomed.2024.12.046

**Published:** 2024-12-24

**Authors:** Anaïs Gardette, Viviana Marzaioli, Samia Bedouhene, Gilles Hayem, Margarita Hurtado-Nedelec, Yantao He, Pham My-Chan Dang, Philippe Dieudé, Zhong-Yin Zhang, Jean-Claude Marie, Jamel El-Benna

**Affiliations:** a INSERM-U1149, CNRS-ERL8252, Université de Paris-Cité, Centre de Recherche sur l’Inflammation, Laboratoire d’Excellence Inflamex, DHU FIRE, Faculté de Médecine, Site Xavier Bichat, Paris, France; b Service de Rhumatologie, Hôpital Bichat, APHP, Paris, France; c Rheumatology Department, Paris-Saint Joseph Hospital Group, Paris, France; d Service d’Hématologie et Immunologie, Hôpital Bichat, APHP, Paris, France; e Department of Medicinal Chemistry and Molecular Pharmacology, Purdue University, 575 Stadium Mall Drive, West Lafayette, IN, 47907, USA; f Molecular Rheumatology, School of Medicine, Trinity Biomedical Sciences Institute, Trinity College Dublin, Dublin, Ireland

**Keywords:** Neutrophils, Lyp, PTPN22, NADPH-Oxidase, Arthritis

## Abstract

Neutrophils are essential for host defense against infections, but they also play a key role in acute and chronic inflammation. The protein tyrosine phosphatase non-receptor type 22 (*PTPN22*) gene encodes the lymphoid-specific tyrosine phosphatase (Lyp) and a genetic single-nucleotide polymorphism of *PTPN22* rs2476601 (R620W) has been associated with several human autoimmune diseases, including rheumatoid arthritis (RA). Here, we investigated the role of Lyp in TNFα–induced priming of neutrophil ROS production and in the development of arthritis using new selective Lyp inhibitors. Results show that Lyp-selective inhibitors (IC-11 and compound 8b), inhibited TNFα-induced priming of neutrophil superoxide anion production. TNFα induced an increase of Lyp protein expression in human neutrophils isolated from healthy donors. Key pathways involved in neutrophil priming were investigated. Lyp-selective inhibitors, inhibited TNFα-induced p47phox phosphorylation on Ser345, ERK1/2 phosphorylation and Pin1 activation. Interestingly, Lyp expression was increased in neutrophils isolated from synovial fluid of RA patients and Lyp inhibitors, I-C11 and compound 8b prevented superoxide anion production by endogenously primed neutrophils isolated from synovial fluid of RA. Moreover, IC-11 significantly prevented collagen antibody-induced arthritis in mice. These results show that Lyp expression is increased in inflammatory neutrophils, Lyp is involved in TNFα-induced excessive ROS production by neutrophils and its inhibition protected mice against arthritis. Inhibition of Lyp could be a therapeutic strategy in inflammatory arthritis.

## 1. Introduction

Neutrophils are key cells of host defense against pathogens, such as bacteria and fungi [1]. During infection or inflammation, circulating neutrophils migrate to the infected tissues where they phagocytose and kill the microbes by producing high quantities of reactive oxygen species (ROS) and releasing proteases and antimicrobial enzymes and peptides [2]. ROS are produced by the phagocyte nicotinamide adenine dinucleotide phosphate (NADPH) oxidase, a multicomponent enzyme system that catalyzes NADPH-dependent reduction of oxygen to superoxide anion $\left({\text{O}}_{2}^{○-}\right)$, a precursor of other ROS [3,4]. In resting cells, the NADPH oxidase system consists of different protein components distributed between the membranes (gp91phox/NOX2, “phox” as phagocyte oxidase, and p22phox which form cytochrome *b*558) and the cytosol (p47phox, p67phox, p40phox, and Rac1/2). Upon stimulation of neutrophils by agonists, such as the bacterial peptide formyl-Met-Leu-Phe (fMLF), the NADPH oxidase components assemble to the membranes where they form the active enzyme complex, which promotes the transfer of electrons from NADPH to molecular oxygen. This activation process is regulated by the phosphorylation of the cytosolic component p47phox [5,6]. Neutrophil NADPH oxidase activation can be potentiated by prior exposure to “priming” agents, such as the pro-inflammatory cytokine tumor necrosis factor alpha (TNFα) [6]. TNFα induces a partial phosphorylation of p47phox on Ser345 by MAPKinases, that is of critical importance for the priming of ROS production in neutrophils [6,7]. Phosphorylated Ser-345 is a binding site for the proline isomerase Pin1 [8]. The binding of Pin1 catalyzes a conformational change of p47phox that facilitates the subsequent phosphorylation of p47phox on other sites and consequently stimulates the activation of the NADPH oxidase.

The *PTPN22* gene encodes for the cytoplasmic lymphoid specific tyrosine phosphatase (Lyp). Lyp is composed of 807 amino acids, its N-terminus contains the catalytic phosphatase domain and its C-terminal contains four proline-rich sequences (P1-P4) [9]. Lyp is expressed solely in hematopoietic cells, it was first identified in lymphoid tissues and mature B and T lymphocytes, and subsequently in myeloid cell lines and in granulocytes [9–11]. Lyp has been shown to be a powerful inhibitor of T-cell and B-cell activation by binding to the SH3 domain of the Csk tyrosine kinase, an important negative regulator of T-cell and B-cell antigen receptor signaling [12–15]. Lyp has gained enormous interest due to a genetic single-nucleotide polymorphism of its gene *PTPN22* rs2476601 (R620W) which has been associated with several human autoimmune diseases, including rheumatoid arthritis (RA) [16–19]. Functional studies of Lyp have been performed mainly in lymphocytes T and B, but its role in neutrophils is less known. Few studies investigated the role of Lyp in human neutrophils, i.e. Bayley et al., showed that the genetic variant R620W of PTPN22 enhanced neutrophil activation [20].

Given the strong association of Lyp with autoimmune diseases and the selective expression of Lyp in immune cells, there is increasing interest in developing Lyp pharmacological inhibitors for the teatment of autoimmune diseases [21,22]. He et al., discovered potent and selective inhibitors of Lyp, called compound IC-11 and 8b [23,24]. Interestingly, these two compounds increased T cell activation and TCR-induced intracellular signaling, supporting their action on Lyp [23–26]. However, the effects of these inhibitors on neutrophil functions and on inflammatory models of diseases such as rheumatoid arthritis (RA) are not known.

Rheumatoid arthritis (RA) is the most common inflammatory arthritis affecting up to 1 % of the world population and is a major cause of disability [27–29]. RA is a systemic inflammatory disease which most commonly targets joints and destroys local articular structures. The pathophysiology of RA involves dysregulated production of inflammatory mediators such as TNFα, T and B cells activation, monocytes/macrophages infiltration in the synovium and polymorphonuclear neutrophils accumulation in synovial fluid [27–29]. The etiology of RA is complex because the factors involved in this pathology can be from different origins such as environmental, immunological or genetic origins. Neutrophils are abnormally present in synovial fluid in RA patients and participate in inflammatory reaction and local articular destruction [30–32]. In synovial fluid, neutrophils are strongly activated by the combination of different stimuli and pro-inflammatory agents such as immune complexes, proteins of complement, pro-inflammatory cytokines (TNFα, IL-8, IL-1β). Activated neutrophils release toxic mediators for tissues such as reactive oxygen species (ROS) and proteases. ROS induce oxidation of proteins, lipids and nucleic acid which contribute to acute and chronic tissue injury in various diseases, including rheumatoid arthritis [33–35]. Indeed, neutrophils are major cells in inflamed synovial fluid of RA patients contributing to joint injury by releasing ROS and proteases.

Studies in the literature have shown that human neutrophils and myeloid cells express Lyp mRNA as compared to other immune cells. Given the key roles of neutrophils and TNFα in RA, we investigated the role of TNFα-Lyp axis in neutrophil ROS production using novel Lyp inhibitors and explored their effect on RA using human synovial fluid and the collagen antibodies-induced arthritis (CAIA) in mice.

## 2. Materials and methods

### 2.1. Reagents

**Fig. 1.**
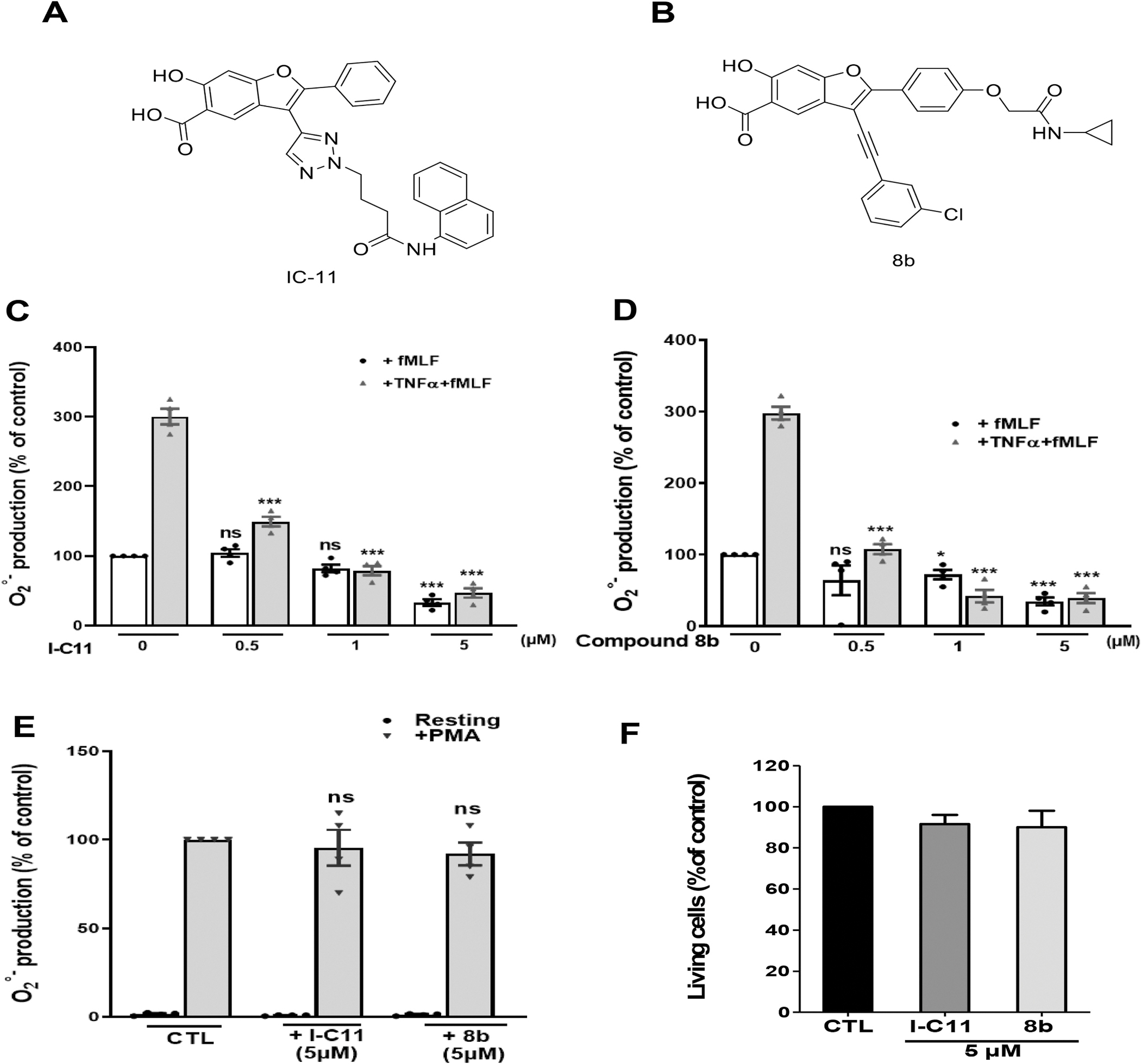
Effect of Lyp inhibitors on superoxide anion production and viability of human neutrophils. Chemical structure of Lyp inhibitors compound IC-11 (A) and compound 8B (B). Neutrophils (1 × 10^6^ cells/ml) were treated with (**C**) increasing concentrations of I-C11 for 15 min, or (**D**) increasing concentrations of 8b for 15 min, in the presence or absence of TNFα for 20 min at 37 °C, then stimulated with fMLF (10^−7^ M). Superoxide anion production was measured using the cytochrome *c* reduction technique. Control corresponds to stimulation with fMLF 10^−7^ M alone: 2.68 ± 0.77 nmol/min/10^6^ cells). **(E)** Neutrophils (1 × 10^6^ cells/ml) were incubated in the absence or presence of I-C11 or 8b for 15 min at 37 °C, then stimulated with PMA (100 ng/ml). Superoxide anion production was measured using the cytochrome *c* reduction technique. **(F)** Neutrophils (1 × 10^6^ cells/ml) were incubated in the absence or presence of I-C11 or 8b for 30 min at 37 °C, then incubated and counted in the presence of 0.1 % Trypan blue. Dead cells were colored in blue, were counted and expressed as % of total cells. Results are presented as mean ± SEM (n = 4, *p < 0.01, **p < 0.001, ***p < 0.0001).

Dulbecco’s phosphate-buffered saline (PBS), bovine serum albumin (BSA), hanks’ balanced salt solution (HBSS), formyl-methionyl-leucylphenylalanine (fMLF), 4-phorbol-12-myristate-13-acetate (PMA), luminol (5-amine-2,3-dihydro-1,4-phtalazinedione), cytochrome *c*, protease and phosphatase inhibitors were from Sigma Chemical Co (Saint Louis, MO, USA). Human TNFα was obtained from PeproTech France (Neuilly Sur-Seine, France). SDS-PAGE (sodium dodecyl sulfate-polyacrylamide gel electrophoresis) and Western blotting reagents were purchased from Bio-Rad (Richmond, CA, USA). The anti-Lyp antibody was from R&D Systems. The anti-phospho-ERK1/2 and anti-phospho-p38MAPK antibodies were from Cell Signaling Technology. Rabbit polyclonal antibodies against p47phox, p22phox and phosphorylated p47phox-sites were generated by our lab as previously described [7,8]. Lyp inhibitors inhibitors IC-11 (Fig. 1A) and compound 8b (Fig. 1B) were prepared as previously described [23–26].

### 2.2. Ethics statement

Venous blood was obtained from healthy volunteers after written informed consent had been obtained. The study was approved by the institutional review boards (IRBs) and ethics committee of INSERM (EFS convention number: 18/EFS/032). All these procedures were conducted in accordance with the 1975 declaration of Helsinki, as revised in 2013.

### 2.3. Human neutrophils, monocytes and lymphocytes isolation

Neutrophils were isolated from venous blood using Polymorphprep gradient centrifugation [36]. The neutrophil band was collected and the cells were washed in PBS and counted. Neutrophils were 96 % pure and 99 % viable. Peripheral blood mononuclear cells (PBMCs) were isolated by Ficoll centrifugation, washed and re-suspended in PBS with 0.5 % bovine serum albumin (BSA) and 1 mM EDTA to a final concentration of 5 × 10^7^ cells/mL. To isolate monocytes and lymphocytes from PBMCs, the cells were subjected to magnetic negative isolation with EasySep^™^ Human Monocyte Isolation, Stemcell Technologies, Vancouver, Canada) (Stemcell) following the manufacturer’s instructions [37]. The average purity of cells using these methods was >96 %.

### 2.4. RNA extraction and polymerase chain reaction

Total RNA was extracted from neutrophils and monocytes and was converted to complementary DNA (cDNA) with the RevertAid H Minus First Strand cDNA Synthesis Kit (Thermo Fisher Scientific Biosciences GmbH) according to the manufacturer’s protocol. End-point PCR was conducted using 1.25 U of Thermo Scientific DreamTaq Polymerase, 0.2 mM dNTPs (Thermo Fisher Scientific Biosciences GmbH), 50 ng cDNA and 0.2 μM of specific primers. The melting temperature was 57 °C for PTPN22 (forward GCCTTGTACTTGGTAGATTGCC, reverse GCAGAAGTTCCTGGATGAGG). Real-time PCR reactions were performed using the Roche LightCycler 480 device and the CliniSciences Kapa Sybr Fast qPCR kit according to the manufacturer’s protocol. Relative expression levels for each gene were calculated using 2^−ddCt^ method with normalization for GAPDH housekeeping gene [37].

### 2.5. Confocal microscopy

Control and TNFα-treated neutrophils were fixed with 2 % paraformaldehyde for 10 min, permeabilized with 90 % methanol for 30 min at 4 °C. Cell suspensions were gently spun by cytospin for 5 min at 300 rpm on glass slides and allowed to dry in air. Cells were then blocked with 1 % BSA in PBS 4 °C, followed by 1 h incubation with mouse anti Lyp antibody, rabbit anti-p22phox antibody or anti-p47phox antibody diluted 1:200. After washing, cells were incubated with Alexa Fluor 555-conjugated goat anti-mouse or Alexa Fluor 488-conjugated goat anti-rabbit antibody (1:200) for 1 h at room temperature in the dark, following by nuclei staining with DAPI (Thermo Scientific) 1:1000 for 10 min in the dark. Slides were then mounted with ProLong antifade reagent. Images were acquired with an inverted confocal laser scanning microscope (Leica SP8, Leica Microsystem) with 40× objective and were analyzed with Leica Microsystem LAS AF software as previously described [37].

### 2.6. Superoxide anion production assay

Neutrophils (10^6^ Cells/ml) were treated with or without Lyp inhibitors for 15 min at 37 °C in HBSS, then incubated in the presence or absence of TNFα. Cytochrome *c* (1 mg/ml) was added and extracellular superoxide anions production was measured in response to fMLF (10^−7^ M) by detecting the superoxide dismutase (SOD)-inhibitable reduction of the ferricytochrome c at 550 nm using Uvikon spectrophotometer [38,39].

### 2.7. Measurement of ROS production by human neutrophils

ROS production was measured by the luminol-amplified chemiluminescence method. Briefly, 5 × 10^5^ human neutrophils were resuspended in 500 μL of HBSS containing 10 μmol/L luminol preheated to 37 °C in the thermostated chamber of the luminometer (Biolumat LB937; Berthold). Neutrophils were incubated in the presence or absence of Lyp inhibitors for 15 min, treated by TNFα (40 ng/ml 15 min) and stimulated by fMLF (10^−7^ M) and chemiluminescence was recorded. Luminol-amplified chemiluminescence was recorded with a luminometer (Berthold-Biolumat LB937). Light emission was expressed in counted photons per minute (c.p.m.) [38,39].

### 2.8. SDS-PAGE and western blot analysis

Neutrophils (15 × 10^6^) in HBSS (400 μL) were treated with Lyp inhibitors and stimulated by TNFα or fMLF at 37 °C with mild shaking. The reaction was stopped by adding 5X concentrated Laemmli sample buffer containing protease and phosphatase inhibitors [40]. Samples were then incubated for 2 min in boiling water and stored at −80 °C until use. Neutrophils lysates were sonicated and subjected to 10 % SDS-PAGE (eq. of 1 × 10^6^ cells/well), using standard techniques. The separated proteins were transferred to nitrocellulose, which was blocked with 5 % milk in Tris-buffered saline containing Tween 20 (TBS-T) for 1 h. After blocking, the membranes were probed with the appropriate anti Lyp antibody (1:2000), or anti-phospho-Ser345-p47phox antibody (1/10 000), or anti-p47phox antibody, or anti-phospho-p38MAPK antibody (1/2000), or anti-phospho-ERK1/2 antibody (1/2000), washed, followed by incubation with HRP-labeled goat anti-mouse antibody (1:10 000) for 1 h at room temperature. After additional washes, the protein bands were revealed by a chemiluminescence method with ECL western blotting reagents and then visualized using a Amersham Imager 600 camera. Alternatively, the membranes were incubated with an alkaline phosphatase-conjugated goat anti-mouse or goat anti-rabbit antibody, and proteins were revealed with NBT/BCIP reagents (Sigma Aldrich, France) in carbonate buffer (100 mM NaHCO_3_, 2 mM MgCl_2_, pH 9.8). Quantification of Western Blot was performed with the software ImageJ.

### 2.9. Pin1 activity assay

Pin1 activity was measured using a previously described technique [8,41]. Neutrophils were incubated in the absence or presence of Lyp inhibitors, treated with TNFα for 15 min and lysed at 10^7^ cells/500 μl in an ice-cold lysis buffer (50 mM HEPES pH7.5, 0.25 % CHAPS, 100 mM NaCl, 1 mM beta-glycerophosphate, 5 mM NaF and 1 mM EGTA) and sonication for 10 s at 4 °C. The assay mixture contains 93 μL HEPES buffer (50 mM HEPES (pH 7.8), 25 μL (60 mg/ml) chymotrypsin solution (Sigma-Aldrich), 6 μL (6 mM) of the peptide Suc-Ala-Glu-Pro-phe-pNA (BACHEM), and 50 μL cell lysate (10^6^ cell equivalent). The absorbance change due to pNA release was followed at 390 nm for 4 min at 10 °C, on the UV–VIS CARY3500 spectrophotometer (Agilent Technologies), results were expressed as OD/min/1million cells.

### 2.10. Animals and evaluation of LYP inhibitor in mice with collagen antibody-induced arthritis

Male BALB/c mice (Janvier Laboratories, Genest-St-Isle, France) of 7 weeks old (25 g) were kept in a standard animal house under normal conditions with 12h light-dark cycle and food and water were provided ad libitum. The animal studies were performed in accordance with the European Community Guidelines. All protocols were approved by the Ethics Committee for Animal Research of University of Paris and INSERM (CEEA-JCM.121). To induce arthritis, mice received intraperitoneally 1.5 mg of a cocktail of monoclonal antibodies against type II collagen (Arthrogen-CIA Arthritogenic Monoclonal Antibody, AMSBIO, UK) on day 0 followed after 3 days by an LPS (50 μg) injection according to described protocol [42,43]. All mice were sacrificed on day 8. Another corresponding series of mice received 100 μl of a solution of 200 μM in 0.5 % DMSO of I-C11 compound intraperitoneally a day before starting the CAIA injection (day 0). A similar dose of I-C11 was administered at day 1, 4 and 6 and these mice also received LPS on day 3. Further, a series of mice (control) did not receive either CAIA or I-C11 compound but only a corresponding injection of physiological saline or 0.5 % DMSO respectively. Mice were weighed and the thickness of hind paws were measured with a digital Vernier caliper (Mitutoyo, Roisy, France). The score for severity of arthritis was measured by observing joints intra-phalangeal, metacarpo-phalangeal and the ankle. Normal joints: score 0, score 1: one of the joints has redness and swelling, score 2: two of the joints have redness and swelling, score 3: All three joints of the joints have redness and swelling and score 4/5for severity of inflammation of the entire paw or hind foot. Two independent experiments were performed with 6 mice per group.

### 2.11. Hematoxylin and eosin staining and MPO immunohistochemistry

The hind paws of sacrificed mice (day 8) were fixed in 4 % formol solution during 48H at room temp. They were decalcified with OSTEOMOLL reagent (Sigma, France) during 5 days and fixed in 4 % formol for 4 H at room temperature, dehydrated and embedded in paraffin. Tissues sections were stained with Hematoxylin and Eosin (H&E) or labeled with polyclonal rabbit anti-human myeloperoxidase (Dako A0398, Agilent, Les Ulis, France) as described previously [44] and quantified using Image J software.

### 2.12. Statistical analysis

All results are expressed as means ± standard error of the mean (SEM). Data were analyzed using GraphPad Prism 7 software (GraphPad Software, San Diego, CA). For two-group comparisons, unpaired t tests were used. One-way ANOVA with a Tukey’s multiple comparison post-test was used for comparisons of more than three groups. *p < 0.05, **p < 0.01, ***p < 0.001, ****p < 0.0001 values are considered significant.

## 3. Results

### 3.1. Lyp inhibitors suppress TNFα–induced priming of superoxide production in human neutrophils

NADPH oxidase-derived superoxide anion and ROS generation is a key inflammatory neutrophil function which can be enhanced or primed by TNFα. To investigate whether Lyp is involved in the stimulation or inhibition of TNFα-induced priming of neutrophil NADPH oxidase, we tested the effect of two different pharmacological Lyp inhibitors, compounds I-C11 (Fig. 1A) and 8b (Fig. 1B). Neutrophils were incubated with Lyp inhibitors and superoxide anions production was assessed by using the cytochrome *c* reduction assay. As expected, TNFα clearly increased fMLF-stimulated superoxide anions production by neutrophils and compounds I-C11 and 8b dramatically inhibited this process even at low μM concentrations (Fig. 1C and D). It is noteworthy that I-C11 and 8b at 0.5 and 1 μM inhibited TNFα–induced priming of superoxide production by human neutrophils without affecting the fMLF-induced response. Interestingly, I-C11 and 8b at the highest concentration used, did not affect PMA-induced superoxide production by neutrophils (Fig. 1E), thus suggesting that their inhibitory effects were due to neither scavenging of superoxide anions nor direct inhibition of NADPH oxidase. Furthermore, viability of neutrophils was not affected at the highest concentration of used inhibitors (5 μM) (Fig. 1F).

To confirm these results, we used another technique which detects several ROS molecules, the luminol-amplified chemiluminescence assays. Results show that I-C11 and 8b inhibited TNFα and fMLF-induced chemiluminescence (Supplemental Fig. S1) and the two inhibitors did not affect PMA-induced ROS production by neutrophils (Supplemental Fig. S2). All together, these results suggest that Lyp activity controls TNFα-induced priming of superoxide anions of ROS production by the phagocyte NADPH oxidase in human neutrophils.

We also tested the effect of Lyp inhibitors on neutrophil degranulation, another TNFα-induced neutrophil key function. The integrin CD11b is mainly localized at the membrane of specific and tertiary granules in neutrophils. Degranulation of these granules is accompanied with fusion of their membranes with the plasma membrane and an increase of the expression of CD11b on the neutrophils surface. Thus, degranulation can be assessed by the detection of CD11b using flow cytometry of nonpermeabilized neutrophils. Results show that fMLF and TNFα induced a clear CD11b expression on the neutrophil plasma membrane and 8b and I-C11 at the highest concentration tested had no effect on this process (Supplemental Fig. S3). These results suggest that Lyp does not control TNFα-induced neutrophil degranulation of specific and tertiary granules.

### 3.2. Lyp protein expression is increased by TNFα in human neutrophils

**Fig. 2.**
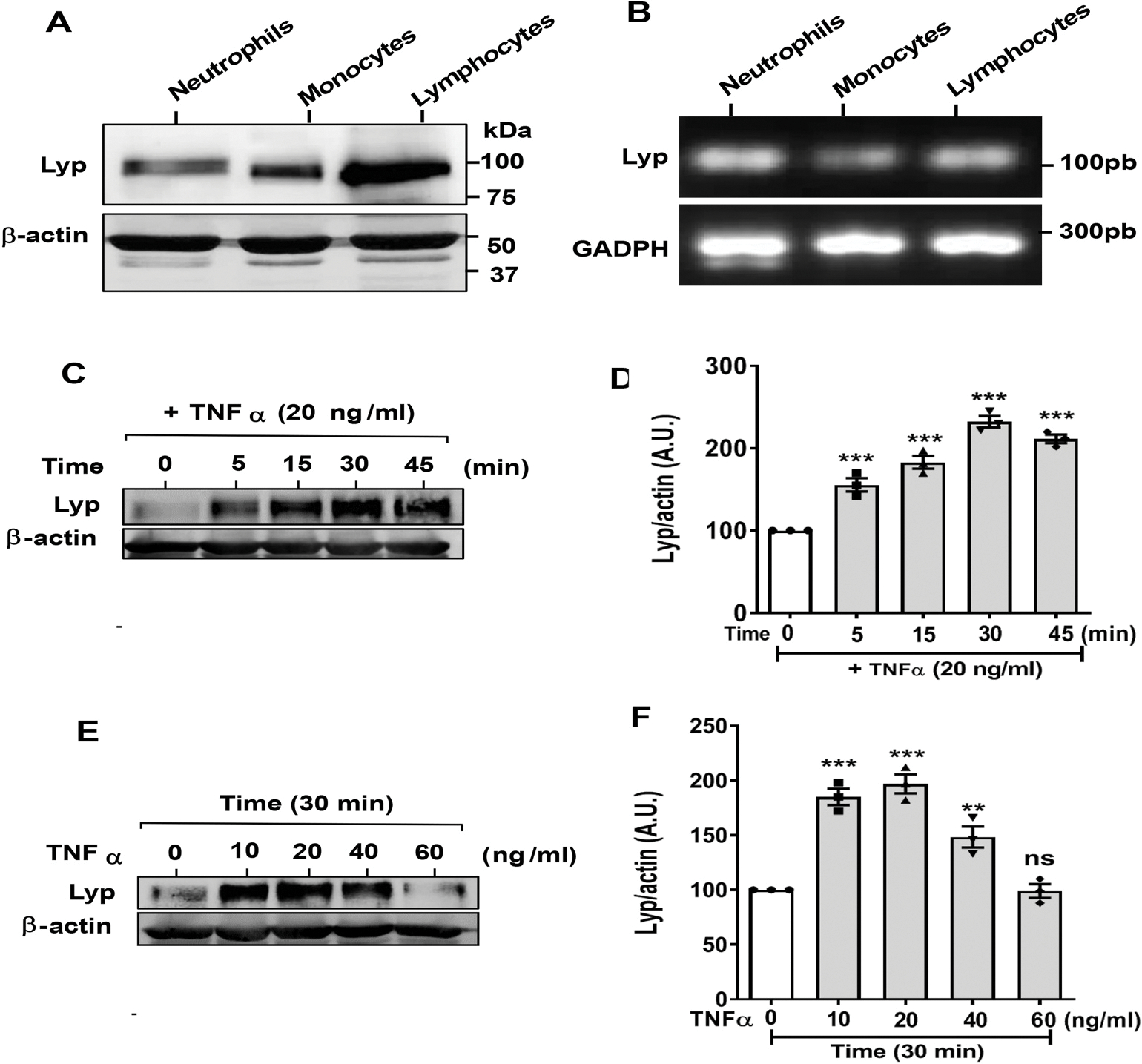
Expression of Lyp in human neutrophils, monocytes and lymphocytes and effect of TNFα on the expression of Lyp in human neutrophils. (**A**) Neutrophils, monocytes and lymphocytes (1 × 10^6^ each), were lysed in Laemmli sample buffer and proteins were analyzed by SDS-PAGE and Western Blot using anti-Lyp antibody and anti-Actin antibody as control. (**B**) Total RNA was extracted from neutrophils, monocytes and lymphocytes (5 × 10^6^ each) using standard techniques. mRNA levels for Lyp were determined by PCR using specific primers. GAPDH was used as loading control. The size of the products is represented in base pairs (bp). (**C**) Neutrophils were incubated in the absence or presence of TNF**α** (20 ng/ml) at 37 °C for different time periods, then lysed in Laemmli sample buffer and proteins were analyzed by SDS-PAGE and Western Blot using anti-Lyp antibody and anti-Actin antibody as control. **(D)** Proteins were quantified and presented as mean ± SEM (n = 4, *p < 0.01, **p < 0.001, ***p < 0.0001). (**E**) Neutrophils were incubated in the absence or presence of TNF**α** (30 min) at 37 °C at increasing concentrations, then lysed in Laemmli sample buffer and proteins analyzed by SDS-PAGE and Western Blot using anti-Lyp antibody and anti-Actin antibody as control. **(F)** Proteins were quantified and results are presented as mean ± SEM (n = 4, *p < 0.01, **p < 0.001, ***p < 0.0001).

As TNFα plays a key role in RA, we wanted to know if this pro-inflammatory cytokine affects Lyp expression in neutrophils from healthy donors. We assessed Lyp expression in different circulating immune cells, human neutrophils, monocytes and lymphocytes (used as a positive control) purified from the same donor, lysed and subjected to SDS-PAGE and Western Blot and mRNA assessment. Results obtained with specific monoclonal anti-Lyp antibody show that circulating untreated human neutrophils clearly express Lyp protein as compared to monocytes and lymphocytes (Fig. 2A). This result was confirmed by the presence of Lyp mRNA in neutrophils, monocytes and lymphocytes (Fig. 2B). Interestingly, the proinflammatory cytokine, TNFα induced a dramatic and rapid increase of Lyp expression in human neutrophils in a time-(Fig. 2C and D) and concentration-dependent manner (Fig. 2E and F). At 20 ng/ml of TNFα, Lyp expression increased after 5 min up to 30 min, then reached a plateau. Lyp expression increased up to 20 ng/ml of TNFα, then decreased at higher concentrations. Such a rapid unusual protein expression in neutrophils has been previously reported for other proteins such as IL-6-receptor and retinoic acid receptor and is known as a signal-dependent translation of constitutive mRNA [45,46]. These results clearly show that the pro-inflammatory cytokine TNFα induced an increase in Lyp protein level in human neutrophils, suggesting an increase of Lyp protein in neutrophils in inflammatory sites.

### 3.3. TNFα induces Lyp colocalization with the NADPH oxidase subunits

**Fig. 3.**
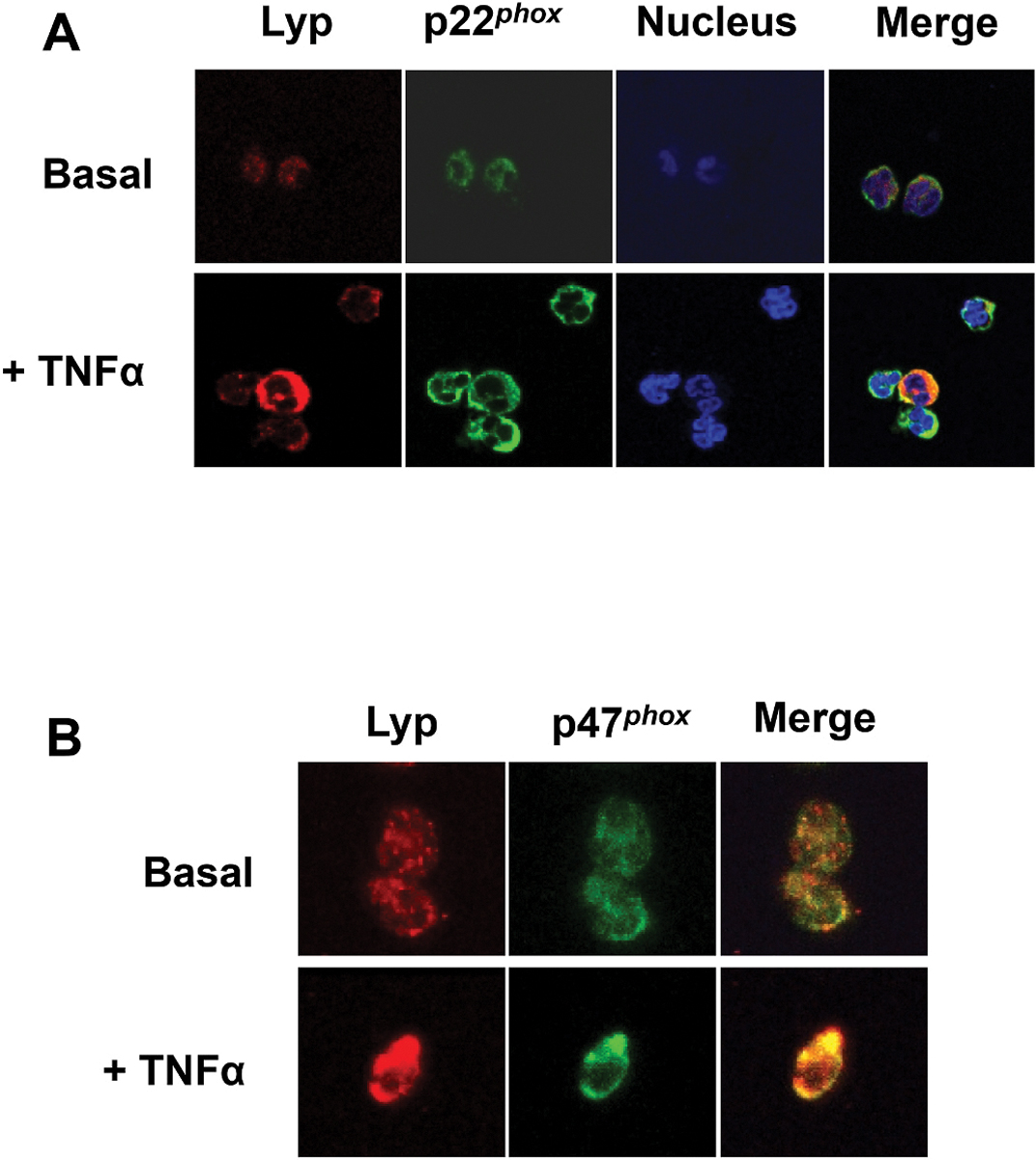
TNFα induced Lyp colocalization with p22phox and p47phox in human neutrophils. Neutrophils were treated with PBS or TNFα (20 ng/ml), and prepared for confocal microscopy. Cells were incubated with a **(A)** rabbit anti-p22phox antibody (1:200) and a mouse anti-Lyp antibody (1:200) or **(B) r**abbit anti-p47phox antibody (1:200) and a mouse anti-Lyp antibody (1:200) followed by incubation with Alexa Fluor 488-(green) conjugated goat anti-rabbit and Alexa Fluor 568 (red) conjugated goat anti-mouse. Stained cells were examined with a confocal microscope and the images were analyzed. (Images are representative of four experiments). (For interpretation of the references to color in this figure legend, the reader is referred to the Web version of this article.)

To investigate how Lyp is involved in TNFα-induced priming of neutrophil NADPH oxidase enzyme complex, we performed confocal microscopy analysis. Results show that TNFα induced the expression of Lyp as compared to basal condition (Fig. 3A and B), confirming the results obtained above with western blots. In resting cells, Lyp weakly colocalized with p22phox and p47phox, the membrane bound and the cytosolic subunit of NADPH oxidase respectively. However, in TNFα-treated neutrophils, Lyp strongly colocalized with both p22phox (Fig. 3A) and p47phox (Fig. 3B), further suggesting that Lyp could be involved in the regulation neutrophil ROS production.

### 3.4. Lyp inhibitors decrease TNFα–induced phosphorylation of p47phox on Ser345, ERK1/2 and activation of Pin1 in neutrophils

**Fig. 4.**
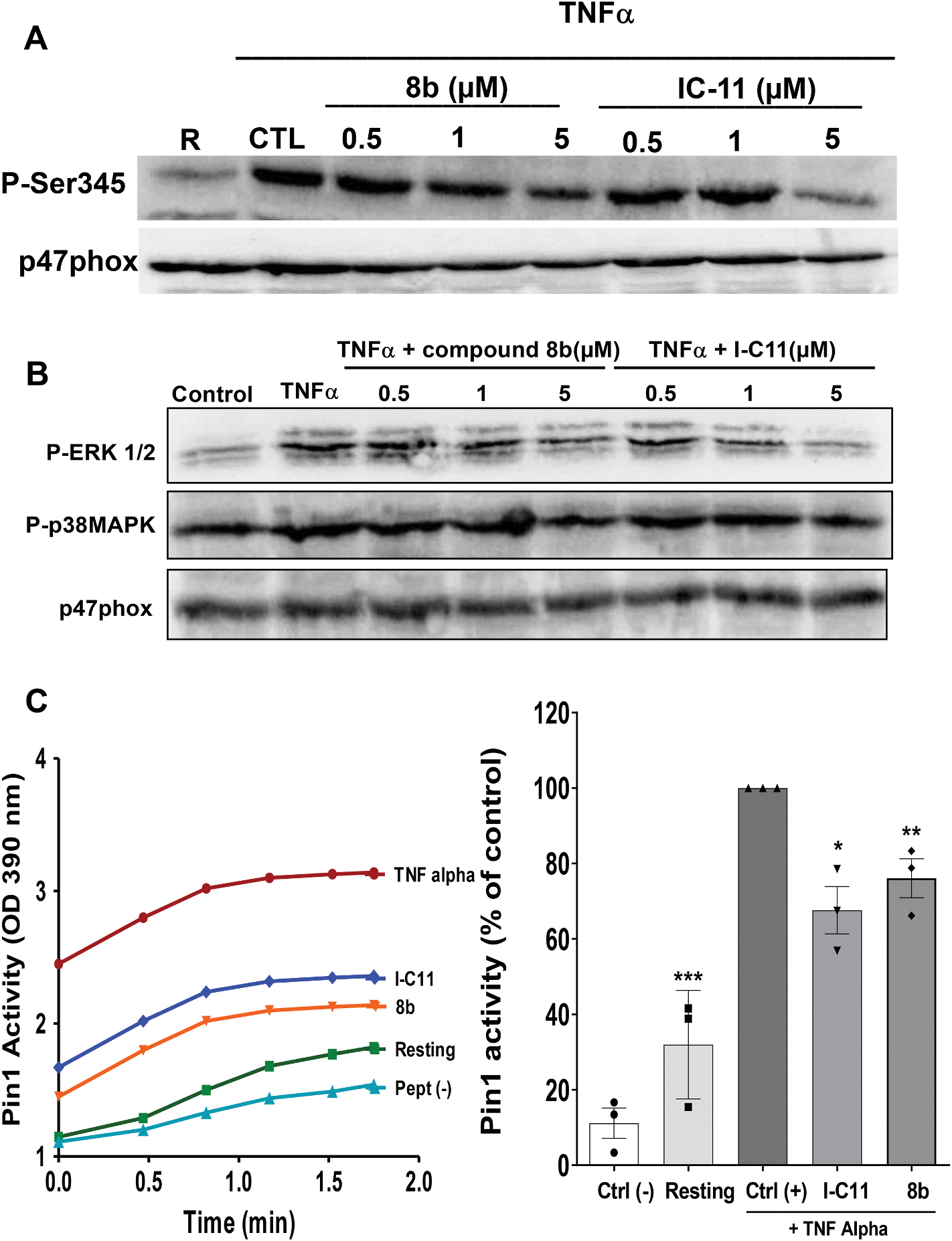
Effect of I-C11 and 8b on TNFα-induced p47phox-serine345 phosphorylation, ERK1/2 and p38MAPK phosphorylation and Pin1 activation in human neutrophils. **(A)** Neutrophils (10 × 10^6^ cells/ml) were incubated with increasing concentrations of I-C11 or 8b in the presence or absence of TNFα for 15 min at 37 °C, then lysed in Laemmli sample buffer and proteins were analyzed by SDS-PAGE and Western Blot using anti-phopho-Ser345-p47phox antibody and anti-p47phox antibody as control. **(B)** or anti-phopho-ERK1/2, anti-phospho-p38MA and anti-p47phox antibody as control. **(C)** Neutrophils (10 × 10^6^ cells/ml) were incubated with I-C11 or 8b in the presence or absence of TNFα for 20 min at 37 °C, then lysed and Pin1 measured as described in the Methods section (left panel) and results are represented as mean ± SEM (n = 3, *p < 0.01, **p < 0,001) (right panel).

To understand how Lyp regulates TNF**α**-induced priming of neutrophil ROS production, we investigated the effect of Lyp inhibitors on TNF**α**-induced phosphorylation of p47phox on Ser345, phosphorylation of ERK1/2 and p38MAPKinase and Pin1 activation, key pathways controlling TNF**α**-induced NADPH oxidase priming [6]. Results show respectively that TNF**α** induced an increase of the phosphorylation of p47phox on Ser345 (Fig. 4A), the phosphorylation of ERK1/2 and p38MAPK (Fig B), and Pin1 activation in human neutrophils (Fig. 4C). Treatment of cells with I-C11 or 8b at 5 μM inhibited the phosphorylation of p47phox on Ser345 (Fig. 4A), the phosphorylation of ERK1/2 and at a lesser extent the phosphorylation of p38MAPK (Fig. 4B), and markedly reduced TNF**α**-induced activation of Pin1 (Fig. 4C). The results obtained suggest a key role of Lyp in TNF**α**-induced priming of ROS production in human neutrophils by controlling MAPKinases activation, p47phox phosphorylation and Pin1 activation.

### 3.5. Lyp expression is increased in neutrophils isolated from synovial fluid of RA patients and lyp inhibitors inhibit their superoxide production

**Fig. 5.**
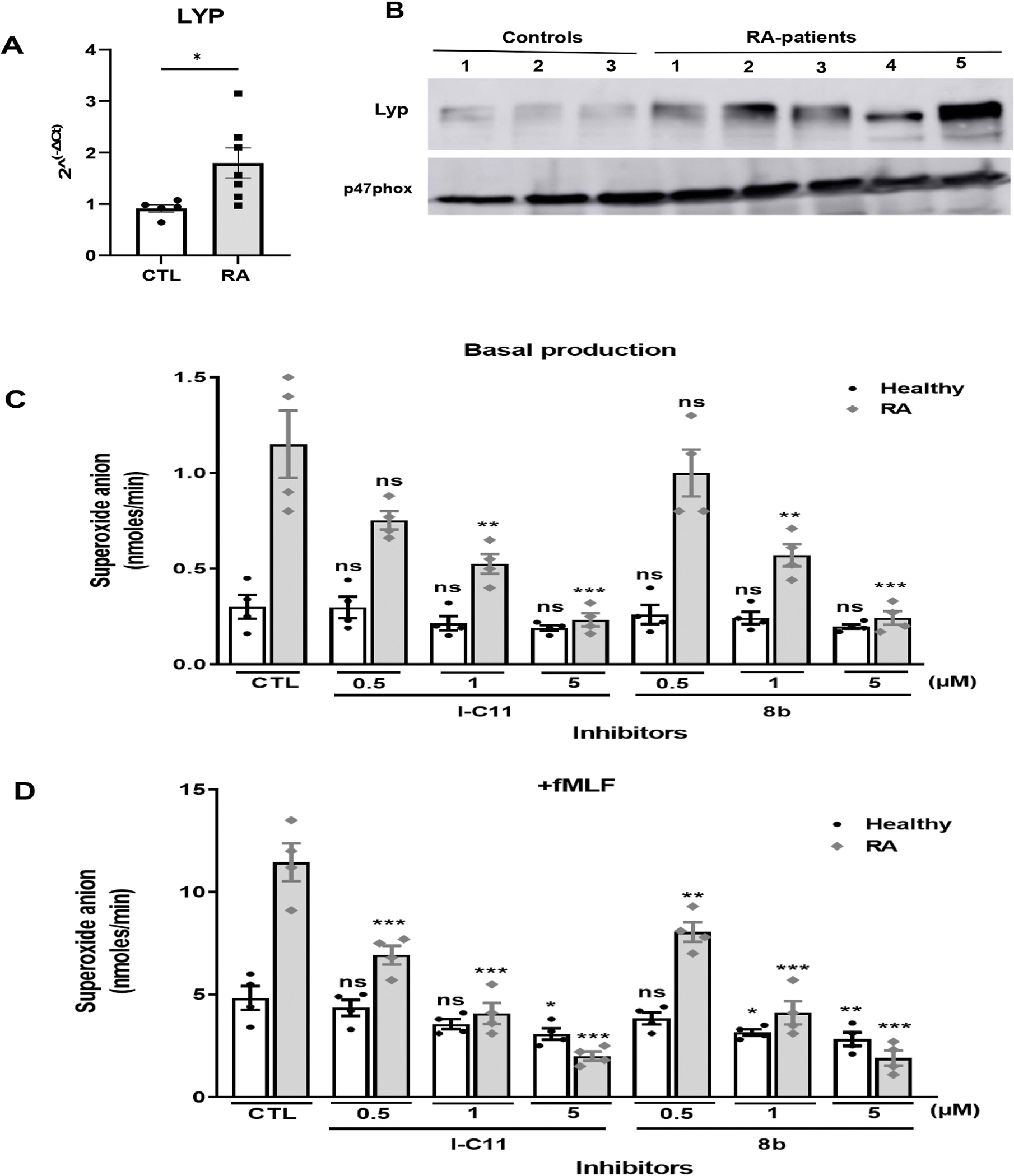
Expression of Lyp in neutrophils isolated from healthy individuals and synovial fluid of rheumatoid arthritis patients and effect of Lyp inhibitors on superoxide anion production. **(A)**Total RNA was extracted from neutrophils (5 × 10^6^ each) using standard techniques. mRNA levels for Lyp were determined by real-time PCR using specific primers. CTL and RA represents healthy individuals and rheumatoid arthritis patients respectively. Results are presented as mean ± SEM (n = 7 RA patient, *p < 0.05) in respect to healthy control (n = 5). (**B**) Neutrophils (1 × 10^6^ each) from 3 controls and 5 RA patients were lysed in Laemmli sample buffer and proteins were analyzed by SDS-PAGE and Western Blot using anti-Lyp antibody and anti-p47phox antibody as control. **(C)** Neutrophils (1 × 10^6^ cells/ml) isolated from synovial fluid of RA patients were incubated with increasing concentrations of I-C11 or 8b for 15 min at 37 °C, then basal superoxide anion production was measured using the cytochrome *c* reduction technique or after fMLF (10^−7^ M) stimulation **(D)**. Results are presented as mean ± SEM (n = 4, *p < 0.01, **p < 0.001, ***p < 0.0001).

Genetic studies have described association of protein tyrosine phosphatase non-receptor type 22 (*PTPN22*) (R620W) polymorphism with RA and other autoimmune diseases. The PTPN22 gene encodes for the cytoplasmic lymphoid specific tyrosine phosphatase (Lyp). The results obtained above with TNFα, suggest that Lyp expression could increase in neutrophils at inflammatory sites. Thus, we investigated Lyp expression in neutrophils isolated from synovial fluid of RA patients as compared with circulating neutrophils of healthy donors. Lyp expression in neutrophils was assessed by quantitative real-time PCR and Western Blot. Results show that Lyp mRNA (Fig. 5A) was significantly increased in neutrophils isolated from RA patients in respect to healthy subjects (CTL). Interestingly, Lyp protein expression was also increased (Fig. 5B), confirming the mRNA results.

Neutrophils isolated from synovial fluid of RA patients are known to be primed and produce a high level of ROS. We therefore tested the effect of two new Lyp inhibitors, I-C11 and compound 8b on superoxide production by neutrophils isolated from synovial fluid of RA patients. The basal superoxide anion production by neutrophils from synovial fluid of RA patients was higher than circulating neutrophils and Lyp inhibitors suppressed this process in a dose-dependent manner (Fig. 5C). Stimulation of neutrophils isolated from synovial fluids of RA patients by fMLF induced more superoxide anion production than control neutrophils (Fig. 5D), clearly showing that neutrophils isolated from synovial fluid of RA patients are already primed. Interestingly, IC-11 and 8b effectively inhibited the superoxide anion production of neutrophils isolated from synovial fluid of these patients after stimulation by fMLF. These results suggest that Lyp phosphatase activity mediates neutrophil hyper-activation in synovial fluid of RA patients and that inhibition of Lyp could dampen this activation.

### 3.6. Lyp inhibitor prevents arthritis development in mice

**Fig. 6.**
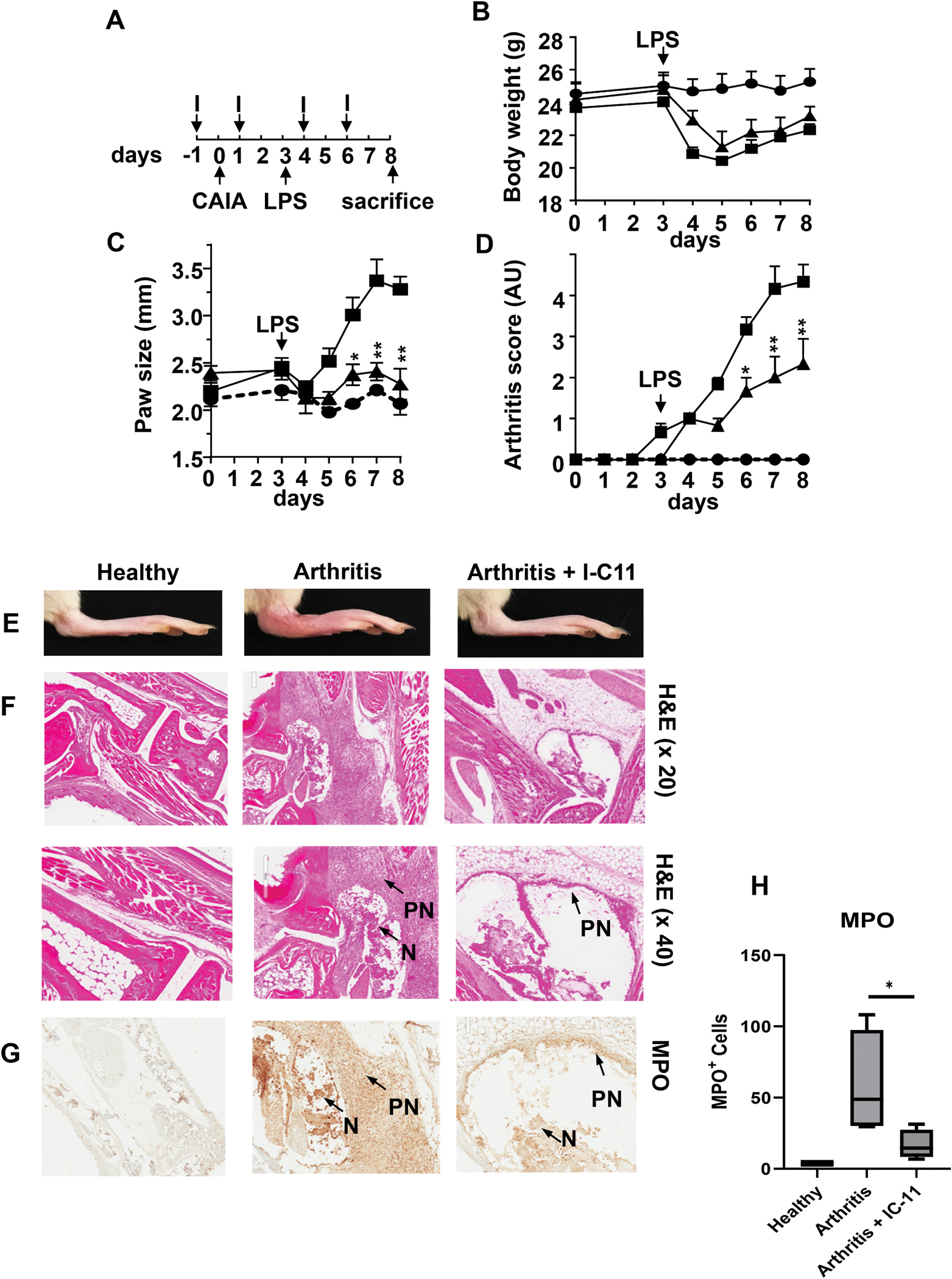
Compound IC-11, a Lyp inhibitor prevents anti-collagen antibodies-induced inflammatory arthritis (CAIA) in mice. CAIA mice was generated by an intra peritoneal injection of anti-type II collagen antibodies on day 0, followed by LPS injection on day 3 **(A)**. Mice without or with IC-11 treatments were compared with control mice which only received physiological saline. The body weight (**B**) the thickness of paws (**C**) and physical arthritis score (**D**) were monitored as described in methods (filled circles correspond to non treated mice; filled squares correspond to mice treated with CAIA and LPS; filled triangles correspond to mice treated with IC-11, CAIA and LPS). Results are mean ± SEM of 2 independent experiments with a total n = 6 mice for each group (*p < 0.05; **p < 0.01; ***p < 0.001). The bottom panels indicate the macroscopic features of the hind legs representative of healthy, arthritic and ICII-treated arthritic mice **(E)**. Further, the hematoxylin and eosin stained paw sections issued from the corresponding mice are shown with a (x20 and ×40) magnification **(F).** Presence of polynuclear cells (PN) and necrcotic damage (N) in magnified inserts are indicated. The tissue was analyzed by immunohistochemistry for myeloperoxidase expression (bottom row) **(G)**. MPO staining was quantified and statistics were performed on 2 independent experiments with a total 6 mice for each group **(H).** Results are presented as mean ± SEM (*p < 0.01).

To investigate if Lyp is involved in arthritis development, we tested the effect of compound IC-11 and 8b on the established collagen antibody induced arthritis (CAIA) mice model. Preliminary results suggested that I-C11 was more effective and was further investigated according to the schematized protocol (Fig. 6A). Results show that the body weights of the mice with or without IC-11 injection decreased similarly after 3 days following LPS injection (Fig. 6B) suggesting no deleterious effect of I-C11. When mice received LPS at day 3, a sharp increase in paw size was observed (Fig. 6C) as compared to control values and this rise was blunted in mice receiving I-C11. Further, the arthritis index score also increased after LPS injection (Fig. 6D) in line with the CAIA mice model. When compound I-C11 was administrated, the arthritis index score was reduced. Mice were sacrificed on day 8 and the representative hind foot of each group is shown in Fig. 6E. Results show that CAIA and LPS-treated mice have a marked swollen hind foot as compared to untreated mice. Interestingly, I-C11 induced a clear reduction in paw swelling of arthritic mice (Fig. 6E). This I-C11 protective effect on arthritis was further sustained by the corresponding histological analysis (Fig. 6F). The H&E staining of hind feet sections showed a normal structure without any cell infiltration for the control or healthy mice (left row). In contrast, arthritic hind feet (middle row) were damaged and associated with steatonecrosis (N) and inflammatory infiltrates composed of mainly polymorphonuclear neutrophils (PN). These arthritic alterations were significantly diminished in IC-11-treated mice. MPO staining of the hind foot tissues confirmed the presence high amount of neutrophils in arthritic mice and I-C11-treated mice had a reduced MPO staining (Fig. 6G and H). Taken together, the results indicate that I-C11, a Lyp inhibitor efficiently protects mice against the development of arthritis.

## 4. Discussion

In this study we examined the role of the phosphatase Lyp in TNFα-induced neutrophil hyper-activation. We show that Lyp inhibitors inhibited TNFα-induced priming of neutrophil superoxide production by inhibiting p47phox phosphorylation on Ser345 and Pin1 activation. We identify a dramatic increase of Lyp expression in human neutrophils in presence of TNFα. Interestingly, Lyp expression was increased in neutrophils issued from synovial fluid of RA patients compared to circulating neutrophils from healthy individuals and that Lyp inhibitors dampen their activation. Also, Lyp inhibitor I-C11 effectively prevented collagen antibody-induced arthritis (CAIA) in mice further sustaining an inflammatory role of Lyp in RA.

Human neutrophils express Lyp protein and mRNA at basal levels compared to other immune cells. Using specific monoclonal antibody, here we show that Lyp protein is expressed in resting neutrophils, monocytes and lymphocytes. Lyp expression was increased in neutrophils isolated from synovial fluid of RA patients, suggesting that pro-inflammatory cytokines, such as TNFα, might be responsible for Lyp upregulation in RA. TNFα, a key pro-inflammatory cytokine in RA was found to induce a dramatic increase of Lyp expression in circulating human neutrophils, thus the increase of Lyp expression in RA could be due to the action of TNFα. TNF**α** induced an unusual rapid protein expression of Lyp in neutrophils, this process has been previously reported for other proteins such as IL-6-Receptor-α [45] and retinoic acid receptor α [46] and is known as a signal-dependent translation of constitutive mRNA. Thus, TNFα could also induce a rapid translation of Lyp mRNA present in neutrophils.

ROS production by neutrophils is believed to induce tissue injury participating to pathological inflammatory processes in RA. TNFα is known to prime neutrophil ROS production involved in RA pathophysiology. Here we found that inhibitors of the phosphatase Lyp (8b and I-C11) at low μM concentrations, inhibited basal and activated superoxide production by synovial fluid neutrophils and TNF-induced neutrophil priming of ROS production. These data suggest that Lyp activity is required for NADPH oxidase up-regulation contrary to its inhibitory role in T and B lymphocytes [12,13]. Indeed, in T lymphocytes, Lyp binds to the protein tyrosine kinase Csk and inhibits TCR signal and inhibitors of Lyp enhanced T cell activation and TCR-induced signaling [10–14]. Our results are in agreement with those obtained by other means, showing that the human genetic variant PTPN22 R620W which induces Lyp gain-of-function [47] enhanced neutrophil ROS production [20] and that deletion of PTPN22 gene in mice inhibited IgG-mediated neutrophil ROS production [48]. Further, we showed that neutrophils isolated from synovial fluid of RA patients express more Lyp protein and are already primed in vivo. Treatment of RA neutrophils with Lyp inhibitors (8b and I-C11), resulted in strong inhibition of both basal and fMLF-induced ROS production. All these data support a positive regulatory role of Lyp in neutrophil ROS production and at the inflammatory sites.

TNFα-induced NADPH oxidase priming in human neutrophils involves two crucial mechanisms, p47phox phosphorylation on Ser345 by MAPKinases such as ERK1/2 and p38MAPK and Pin1 activation [7,8]. Here we show that Lyp inhibitors significantly inhibited TNFα-induced p47phox phosphorylation on Ser345, TNFα-induced ERK1/2 and p38 phosphorylation and Pin1 activation at 5 μM. How Lyp controls these pathways will be investigated further.

Excessive presence of neutrophils is often associated with several inflammatory diseases and can represent 90 % of leucocyte in RA joint [30–34]. Thus, it was of further interest to test Lyp inhibitors in an in vivo model of experimental arthritis. Interestingly Lyp inhibitors were able to prevent arthritis induced in mice by CAIA antibodies further supporting a role of Lyp in RA in human. Despite the stimulatory effect of Lyp inhibitors on T lymphocytes in vitro [23–26], they have a beneficial effect on inflammation in vivo, probably by inhibiting excessive neutrophil activation and release of toxic agents that participates to the inflammatory reaction. This result agrees with the results of Vermeren et al. [48] and Sood et al. [49] who reported that deletion of the PTPN22 gene in mice reduced immune complex-mediated arthritis and mannan-induced autoimmune arthritis respectively. Also, it was reported that Lyp controls protein citrullination, a process related to RA in human [50] Interestingly, the W620R polymorphism in PTPN22 gene disrupts its interaction with peptidylarginine deiminase type 4 and enhances citrullination of proteins. Since Lyp is solely expressed in immune cells, inhibition of Lyp could be a good pharmacological strategy to treat immune-inflammatory diseases without having unwanted toxic effects on other organs. Developing a safe and tolerated Lyp inhibitors for human could be of interest for the treatment of RA.

In conclusion, our results show that Lyp expression was increased in inflammatory neutrophils in RA, TNFα was able to induce a dramatic increase of Lyp expression in human neutrophils, Lyp activity is required for NADPH oxidase priming of ROS production and Lyp inhibitors protect mice from arthritis. Further studies are necessary to identify Lyp partners in neutrophils and to understand how Lyp controls p47phox phosphorylation and Pin1 activation in neutrophils. This study highlights the key role of Lyp in TNFα induced neutrophil hyper-activation, and suggests that Lyp inhibition could be a potential strategy to inhibit neutrophil-related inflammation in RA and other inflammatory diseases.

## Supplementary Material

Supplemental info

## Appendix A. Supplementary data

Supplementary data to this article can be found online at https://doi.org/10.1016/j.freeradbiomed.2024.12.046.
