# Supplementary material for: The protein tyrosine phosphatase Lyp/PTPN22 drives TNFα-induced priming of superoxide anions production by neutrophils and arthritis
Source: Free Radic Biol Med. Author manuscript; Available in PMC 2026 Mar 16. (PMC12990828; doi:10.1016/j.freeradbiomed.2024.12.046)
Supplement: Supplemental info [file NIHMS2139847-supplement-Supplemental_info.docx]

**Supplementary information (SI) for the manuscript:**

**The protein tyrosine phosphatase Lyp/PTPN22 mediates TNFα-induced neutrophil NADPH oxidase priming and its inhibition protects mice from arthritis**

**Anaïs Gardette^1,2^, Viviana Marzaioli^1,6^, Samia Bedouhene^1^, Gilles Hayem^3^, Margarita Hurtado-Nedelec^1,4^, Yantao He^5^, Pham My-Chan Dang^1^, Philippe Dieudé^1,2^, Zhong-Yin Zhang^5^ , Jean-Claude Marie^1^ and Jamel El-Benna^1*^**

***Corresponding author:** [jamel.elbenna@inserm.fr](mailto:jamel.elbenna@inserm.fr)

**This PDF file includes:**

Figure S1 to S3.

**Lyp inhibitors suppressed TNFα+fMLF-induced ROS production in human neutrophils as measured by luminol-amplified chemiluminescence assay**

**Figure S1. Effect of Lyp inhibitors on ROS production by TNFα+fMLF-activated human neutrophils.** Neutrophils (1 x 10^6^ cells / ml) were treated with (**A**) increasing concentrations of I-C11 for 15 min, or (**B**) increasing concentrations of 8b for 15 min, in the presence of TNFα for 20 min at 37°C, then stimulated with fMLF (10^-7^ M). ROS production was measured using luminol-amplified chemiluminesce. The experiment was repeated 3 times.

**Lyp inhibitors did not affect PMA-induced ROS production in human neutrophils as measured by luminol-amplified chemiluminescence assay**

**Figure S2. Effect of Lyp inhibitors on ROS production by PMA-stimulated neutrophils.** Neutrophils (1 x 10^6^ cells / ml) were treated with 5µM of I-C11 or 8b for 15 min, then stimulated with PMA (100 ng/ml). ROS production was measured using luminol-amplified chemiluminesce (Upper panel). **(B)** The experiment was repeated 4 times and quantified (Lower panel).

**Lyp inhibitors did not affect neutrophil degranulation as measured by CD11b plasma membrane expression**

**Figure S3. Effect of Lyp inhibitors on neutrophil degranulation.** Neutrophils (1 x 10^6^ cells / ml) were treated with 5µM of 8b or I-C11 for 15 min, stimulated by fMLF or TNFα for 20 min at 37°C. Plasma membrane CD11b was then detected using a specific antibody and a labeled secondary antibody and flow cytometry technique. The experiment was repeated 3 times.
